# A method of predicting the *in vitro* fibril formation propensity of Aβ40 mutants based on their inclusion body levels in *E*. *coli*

**DOI:** 10.1038/s41598-019-39216-z

**Published:** 2019-03-06

**Authors:** Kalyani Sanagavarapu, Elisabeth Nüske, Irem Nasir, Georg Meisl, Jasper N. Immink, Pietro Sormanni, Michele Vendruscolo, Tuomas P. J. Knowles, Anders Malmendal, Celia Cabaleiro-Lago, Sara Linse

**Affiliations:** 1 Lund University, Biochemistry and Structural Biology, Chemical Center, Lund, Sweden; 2 GEPADO-GmbH, Dresden, Germany; 3 Department of Integrative Structural and Computational Biology, The Scripps Research Institute, 10550 N, Torrey Pines Road, La Jolla, CA 92037 USA; 4 University of Cambridge, Chemistry Department, Lensfield Road, Cambridge, UK; 5 Lund University, Physical Chemistry, Chemical Center, Lund, Sweden; 6 Cavendish Laboratory, Department of Physics, University of Cambridge, JJ Thomson Avenue, Cambridge, UK; 7 Present Address: Faculty of natural sciences, Kristianstad University, Kristianstad, Sweden

## Abstract

Overexpression of recombinant proteins in bacteria may lead to their aggregation and deposition in inclusion bodies. Since the conformational properties of proteins in inclusion bodies exhibit many of the characteristics typical of amyloid fibrils. Based on these findings, we hypothesize that the rate at which proteins form amyloid fibrils may be predicted from their propensity to form inclusion bodies. To establish a method based on this concept, we first measured by SDS-PAGE and confocal microscopy the level of inclusion bodies in *E*. *coli* cells overexpressing the 40-residue amyloid-beta peptide, Aβ40, wild-type and 24 charge mutants. We then compared these results with a number of existing computational aggregation propensity predictors as well as the rates of aggregation measured *in vitro* for selected mutants. Our results show a strong correlation between the level of inclusion body formation and aggregation propensity, thus demonstrating the power of this approach and its value in identifying factors modulating aggregation kinetics.

## Introduction

Alzheimer’s disease (AD) is associated with abnormalities in protein folding, resulting in the misfolding and aggregation of a characteristic set of proteins^1–4^. In particular, the major components of the amyloid plaques found in the brain tissue of AD patients are Aβ peptides, which are derived by proteolysis from amyloid precursor protein (APP)^5–8^. Aβ peptides of different lengths are formed in the brain by proteolytic cleavage of APP by the β- and γ-secretases^9–13^. Since the concentration of the Aβ40 peptide is higher than that of any other amyloid peptides in the aggregates found in human brains^14^, it is important to understand the molecular driving forces for aggregation of this major Aβ form.

A powerful approach to obtain such understanding is to perform the systematic kinetic analysis of a series of designed mutations of the Aβ sequence combined with changes in the solution composition^15–17^. The results of such studies indicate that the aggregation of unstructured Aβ monomers into highly ordered amyloid fibrils is a complex process that is influenced by a vast number of factors. This includes intrinsic factors such as charge, hydrophobicity and other amino acid properties^18^, as well as extrinsic factors like pH, temperature and ionic strength^16,19–22^.

A related strategy for understanding the driving forces for Aβ40 aggregation is to carry out random mutagenesis coupled to an *in vivo* assay, the readout of which may correlate with aggregation propensity. This strategy can be implemented by performing overexpression of recombinant Aβ40 in *E*. *coli*, which is widely used as an affordable source of human proteins. The recombinant proteins can be found either in the soluble or insoluble fraction, the latter usually appears in the form of inclusion bodies. Inclusion bodies contain misfolded proteins of β-sheet structure^23,24^ and can typically interact with β-sheet binding dyes such as Congo red and thioflavin-T (ThT), which are also used to identify and characterize amyloid fibrils^25,26^. The presence of aggregates with amyloid fibril morphology has been observed for inclusion bodies of *E*.*coli* expressing Aβ peptide shown by electron microscopy and AFM^27^. Inclusion body formation has been exploited for purification of several recombinant proteins, because of the ease of isolation from the majority of soluble *E*. *coli* proteins by repeated sonication and centrifugation^28^. While expression of soluble short unstructured peptides and proteins may lead to rapid proteolysis and poor yield, the formation of inclusion bodies protects peptides from degradation and enables the tag-free expression of Aβ peptides^29^.

**Figure 1:**
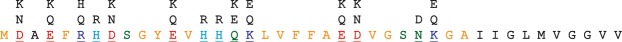
Amino acid sequence of wild-type Aβ40 (colored until residue number 30 based on amino acid properties). The 24 single amino acid mutations analyzed in this work are also shown (in black).

Several studies have demonstrated a connection between formation of inclusion bodies and the propensity to form amyloid fibrils^25,27,30–32^. Here we ask whether such a correlation can be utilized to build a screening method for identification of Aβ40 peptide variants with increased or decreased aggregation propensity. An earlier method using green fluorescent protein fused to Aβ as a reporter of Aβ misfolding made it possible to screen for variants with decreased aggregation propensity only^33^. We also investigate if there is a correlation between the amount of inclusion bodies formed and measures of overall aggregation rate *in vitro*, such as the time it takes until the reaction is half completed, the steepness of the transition or the concentration dependence of the reaction. We use an Aβ40 mutant library of the wild-type protein and 24 single mutation variants (Fig. 1 and see Supplementary Table S1), in which we have changed all charged positions to oppositely charged or neutral hydrophilic amino acid residues, and all His and Asn residues to charged ones. The rational for this design comes from earlier studies that indicated that electrostatic interactions strongly affect the aggregation kinetics of Aβ peptides^16,18,20,22,34^. Using a charge mutant library should thus provide a benchmark for an inclusion-body-based screening method.

## Results

### Analysis of Aβ40 charge mutants expression profiles

**Figure 2:**
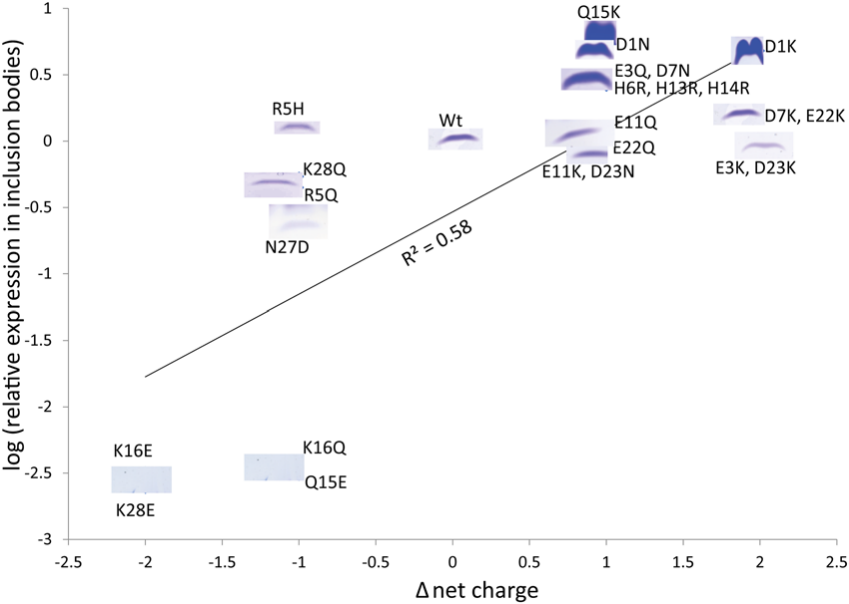
Expression profile of Aβ40 charge mutants in inclusion bodies (y- axis has log values of relative score obtained from imageJ analysis of SDS-PAGE gel bands of mutants) *versus* the change in net charge relative to the wild-type peptide. Supplementary Fig. S3 shows uncropped SDS-PAGE gels of the Aβ40 charge mutant bands included here.

Based on the amino acid sequence, the wild-type Aβ40 peptide has a net charge between −3 and −4 at neutral pH. After successful expression and cell disruption of several mutants, the soluble fraction and inclusion bodies of each mutant were analyzed by SDS-PAGE to evaluate the Aβ40 expression profile (monomeric band at 4.5 kDa). A clear variability in the amount of inclusion bodies was observed between the mutants and wild-type Aβ40. No expression in the soluble fraction could be detected by SDS-PAGE analysis, this is likely a combination of rapid formation of inclusion bodies by the aggregation-prone Aβ40 and because the peptide is relatively small and unstructured and thus gets degraded in *E*. *coli* if not protected in the form of inclusion bodies. As shown in Fig. 2, there is a clear correlation between the amount of inclusion bodies formed and the peptide net charge. The mutants that form a high amount of inclusion bodies have a positive change of the net charge towards neutral (top right corner of Fig. 2). In contrast, the mutants that form a low amount of inclusion bodies have an increased net negative charge (bottom left corner of Fig. 2). A few mutants with inclusion body levels similar to the wild-type are seen close to the center of the plot (Fig. 2).

Of the mutants with one unit less negative charge (i.e. substitution of a negatively charged amino acid by a neutral one or a neutral amino acid by a positively charged one) D1N, Q15K, H6R, D7N, H13R, and H14R formed more inclusion bodies than wild-type. Especially Q15K, but also D1N, stand out as mutants with massive inclusion body formation. Of the mutants with two units less negative net charge, especially D1K but also D7K, form much more inclusion bodies than the wild-type Aβ40. However, E3K, E11K, E11Q and D23K do not; E3K shows lower amounts of inclusion bodies while the other three form similar amounts of inclusion bodies as the wild-type peptide. These results imply that net charge is not the only determinant of inclusion body formation, but that also the position of the charge change and other properties matter. For most of the Aβ40 mutants for which the net charge is decreased (i.e. becomes more negative), a decrease in the level of inclusion body formation is observed. The mutants that showed lower amounts of inclusion bodies include N27D and K28Q, both of which are one unit more negatively charged. Furthermore, some of the more negative mutants failed to form any inclusion bodies using the overnight expression protocol. This includes K16Q and Q15E, both of which are one unit more negatively charged and K16E which is two units more negatively charged.

Taken together, these results show that the level of inclusion body formation depends strongly on peptide net charge, but that it is also affected by the position of the substitution and changes in other properties that accompany the charge change.

To explore the influence of other parameters, we calculated the correlation coefficients between amount of inclusion bodies as judged on a score from imageJ and the mutation induced change in a number of parameters, including charge, helix propensity, β-sheet propensity, hydrophobicity and a computationally predicted aggregation propensity (CamSol score)^35,36^ (Table 1) Camsol is a general algorithm that can be used for any peptide variant and we are testing if it provides a correlation with observed aggregation propensity.

**Table 1:** Correlation(R) between the logarithmized inclusion body levels and several quantitative descriptors for all 24 charge mutants and wild-type Aβ40, and, separately, for mutants that are less (16) and more (7) negative than the wild-type.

Parameter	All	Less negative	More negative
charge	0.76	−0.21	0.59
α-helix propensity	0.49	0.23	0.90
β-sheet propensity	−0.33	−0.08	0.26
hydrophobicity	−0.04	0.29	−0.52
CamSol score	0.75	−0.41	0.64
linear component	0.81	−0.11	0.82

We found a weak correlation between inclusion body formation level and charge when considering all mutants (R^2^ = 0.57), but less so when looking at the less or more negatively charged mutants separately, especially for the less negative mutants that show a slightly negative correlation with charge. Thus, it seems like the direction of the change relative to wild-type is more important than the amplitude of the change. Furthermore, the correlation with changes in helix propensity is very strong for the more negatively charged mutants. This doesn’t imply that the mutants adopt a helical conformation but reflects the properties of the amino acids. We note that the substitutions Q to E, K to Q and K to E reduce the helical propensity while N to D increases it.

**Figure 3:**
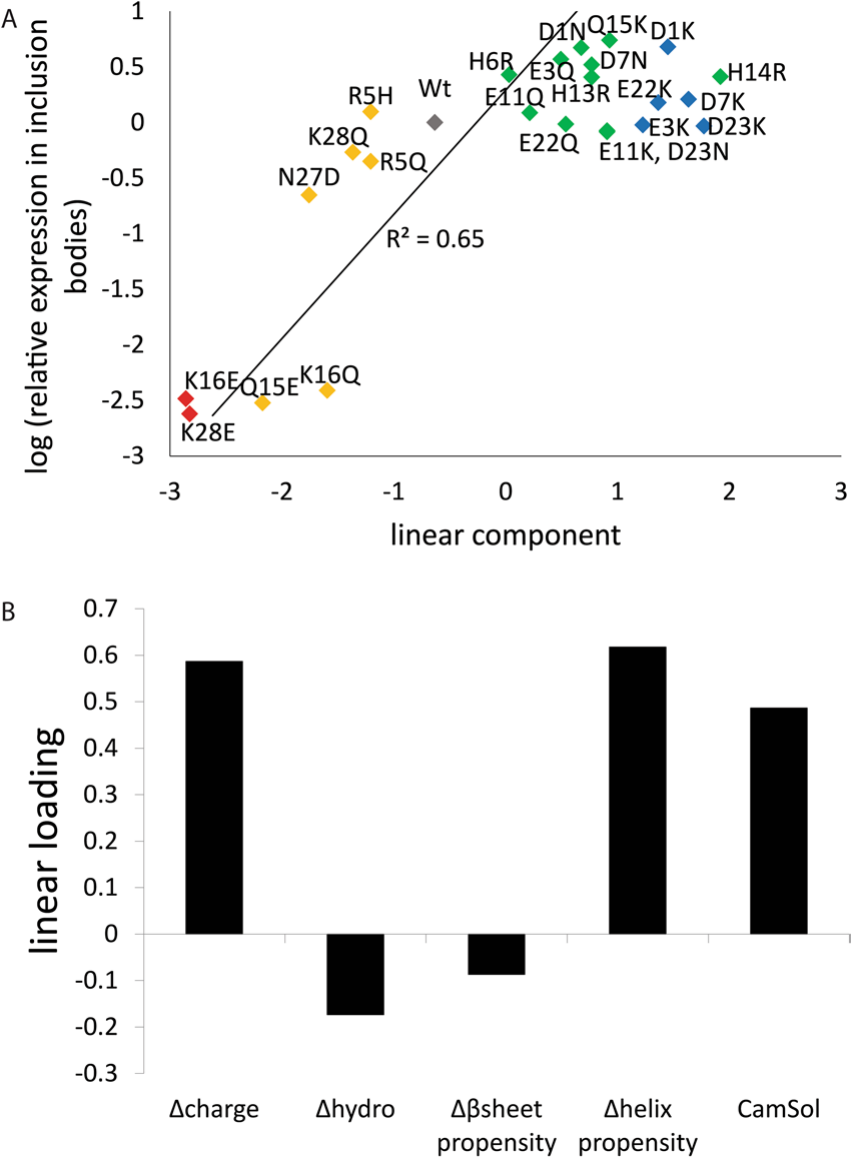
Linear principal component including all quantitative parameters. Scores vs inclusion body levels (A) and loadings (B). The symbols are colored as per the net charge difference relative to wild-type peptide (blue: 2 units, green: 1 unit, grey: 0, yellow: −1 unit, red: −2 unit).

In order to obtain a more detailed overview of the dependence of the inclusion body formation level on the different sequence dependent properties affected by the mutations, a principal component analysis (PCA) was performed on the mutation-induced changes in charge, α-helix propensity, β-sheet propensity, hydrophobicity and CamSol score. The four resulting principal components, describing 99.8% of the variation in these five parameters, were rotated in order to find the component that showed the most linear relationship with inclusion body formation level (Fig. 3). This linear component (Fig. 3A) explains 48% of the variation of the 5 properties. The overall correlation of the linear component is higher than for any of the 5 individual properties and it shows a strong correlation also if the more negatively charged mutants are considered separately (Table 1).

The plots of the loading (Fig. 3B) show to what extent the different parameters contribute to the linear component. Notably, all properties except β-sheet propensity give a positive contribution and charge has the most important contribution.

These results show that the sign of the change in charge is the most important factor for determining the level of inclusion body formation, that it is also dependent on other factors, and that the less negative and more negative mutants may be sensitive to different factors.

### Confocal laser scanning microscopy (CLSM) imaging

**Figure 4:**
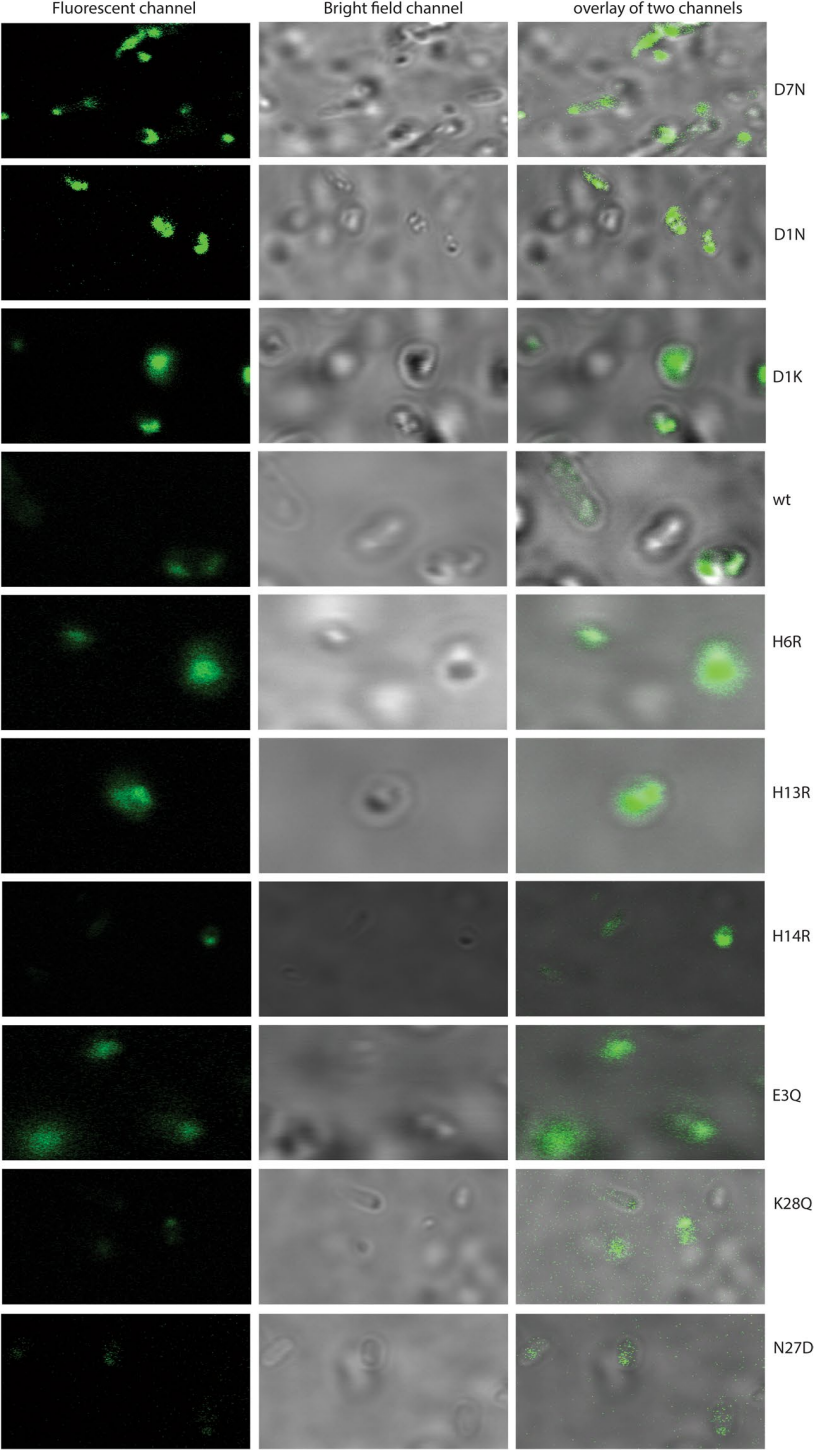
Confocal microscopy images of ThS stained inclusion bodies localized in live *E*. *coli* cells expressing Aβ40 charge mutants and the corresponding bright field images.

We used CLSM to test whether the variations in inclusion body formation of different mutants in bacterial cells is observable at the single-cell level by comparing images obtained from samples prepared in an identical manner (Fig. 4). The mutants selected for aggregation kinetic studies (see below) were imaged. The cell-permeable dye thioflavin S (ThS) was used as a reporter on inclusion bodies, which were observed in the fluorescent channel (green color) with bright field images showing the outline of bacterial cells and slightly darker inclusion bodies (Fig. 4). Live bacterial cells expressing the mutants showed variations in the intensity of fluorescence and can be broadly classified in three classes relative to the wild-type in inclusion body level - higher (D1K, D1N and D7N), similar (E3Q, H6R, H13R and H14R) and lower (K28Q and N27D). The observed pattern agrees with the levels determined by SDS-page analysis (Fig. 2). Detailed classification is limited with this technique as intensities can depend on minor details like sample depth due to scattering effects, laser instability or optics oscillation, slight changes in the angle of coverslip on the sample, etc. While many factors contribute to the recorded intensity, a striking trend is observed that underlines the results found in our other experiments.

### *In vitro* aggregation kinetics of selected mutants

**Figure 5:**
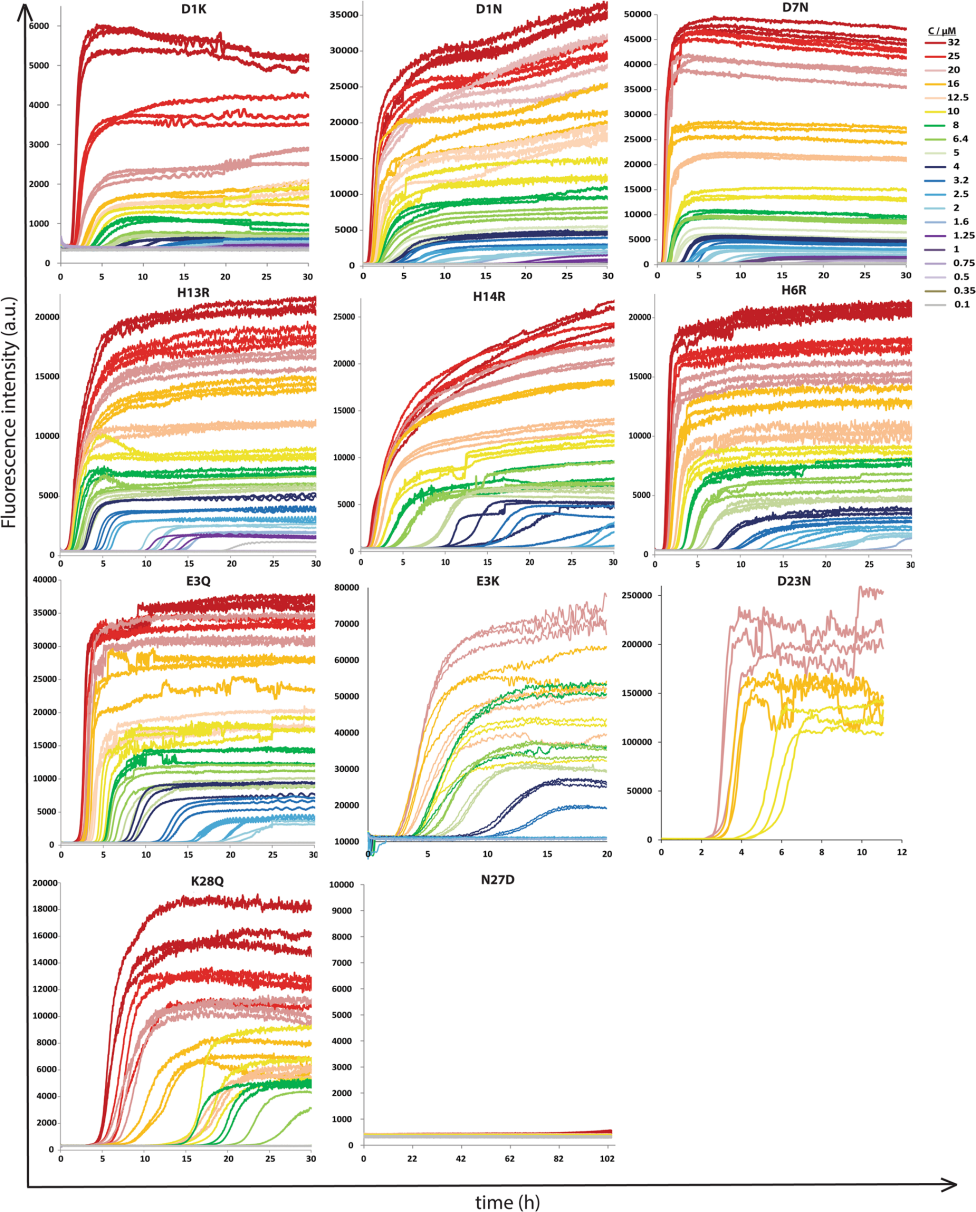
Aggregation kinetic curves of Aβ40 charge mutants (D1K, D1N, D7N, H13R, H14R, H6R, E3Q, E3K, D23N, K28Q and N27D) at a concentration range of 0.1 µM to 32 µM. Aggregation was followed for 30 h except for N27D where it was followed for 102 h. Note: Y-axis scale is not the same because of variation in ThT intensity.

**Figure 6:**
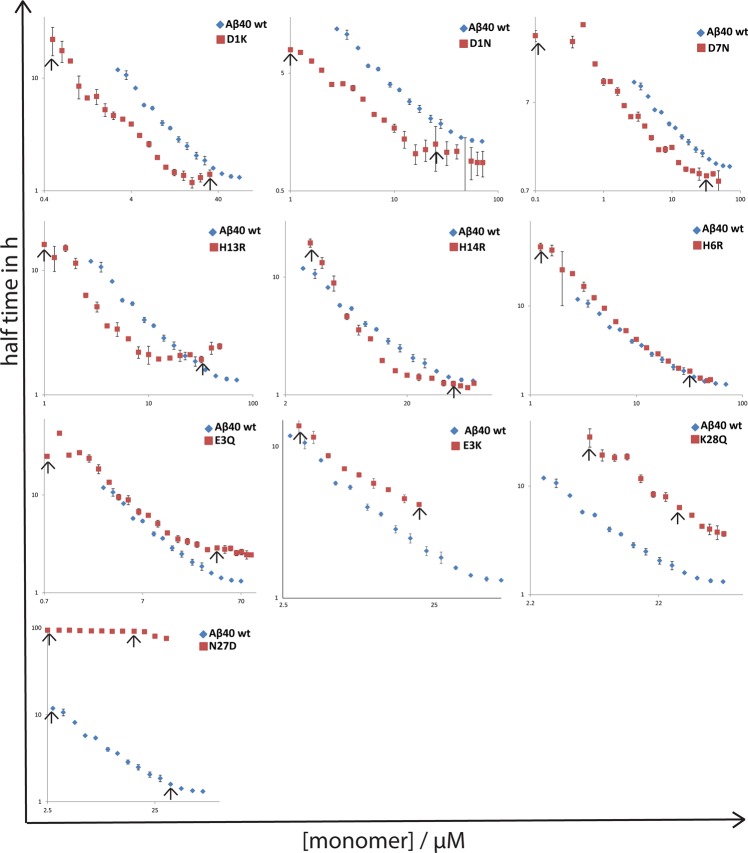
Comparison of t_1/2_ of aggregation for different variants [Aβ40 wild-type, D1K, D1N, D7N, H13R, H14R, H6R, E3Q, E3K, K28Q and N27D] vs. concentration. Log-log plots of faster fibril forming mutants (D1K, D1N, H13R, H14R and D7N) lie below the Aβ40 wild-type curve while the slower fibril forming mutant (E3K, K28Q and N27D) show its curve above the wild-type. H6R and E3Q almost merge with the Aβ40 wild-type. Data points between the arrows represent the half time points of concentrations 32 µM (E3K – 20 µM) to lowest possible. D23N is not included as we have half times available only for three different concentrations.

The propensities of Aβ40 mutants to form fibrils were assessed by monitoring changes in ThT fluorescence intensity for purified and initially monomeric peptides. This approach relies on the specific property of ThT to undergo an intensity increase of its fluorescence emission due to appearance of a new excitation peak close to where we excite upon binding to amyloid fibrils^37^. *In vitro* aggregation kinetics was studied for eleven of the 24 peptide variants, which were chosen to be representative of those showing higher and lower amounts of inclusion bodies compared to the wild-type Aβ40, as well as mutants having similar levels of inclusion bodies to wild-type Aβ40. The aggregation kinetics of these mutants was monitored at a set of different monomer concentrations the lowest being 0.1 µM in each case and the highest in the range 30–89 µM depending on what concentration could be achieved after purification. To facilitate the comparison of the rate of fibril formation of each mutant, the raw data for all mutants were plotted over a fixed range of monomer concentrations (Fig. 5), from 0.1 to 32 µM. However, half-time plots over the whole concentration range sampled are shown in Fig. 6. All mutants except N27D showed sigmoidal-like ThT fluorescence curves with a clear lag phase, a transition and a plateau over most of the concentration range studied (above ca. 2–3.5 µM), indicating that fibrils were formed in most of the samples during the time-frame of the experiment (up to 27 h). The N27D mutant seems to have reached the end of the lag phase after ca. 40–100 h, at concentrations of 32–71 µM, whereas at lower concentrations no increase in fluorescence was observed.

**Figure 7:**
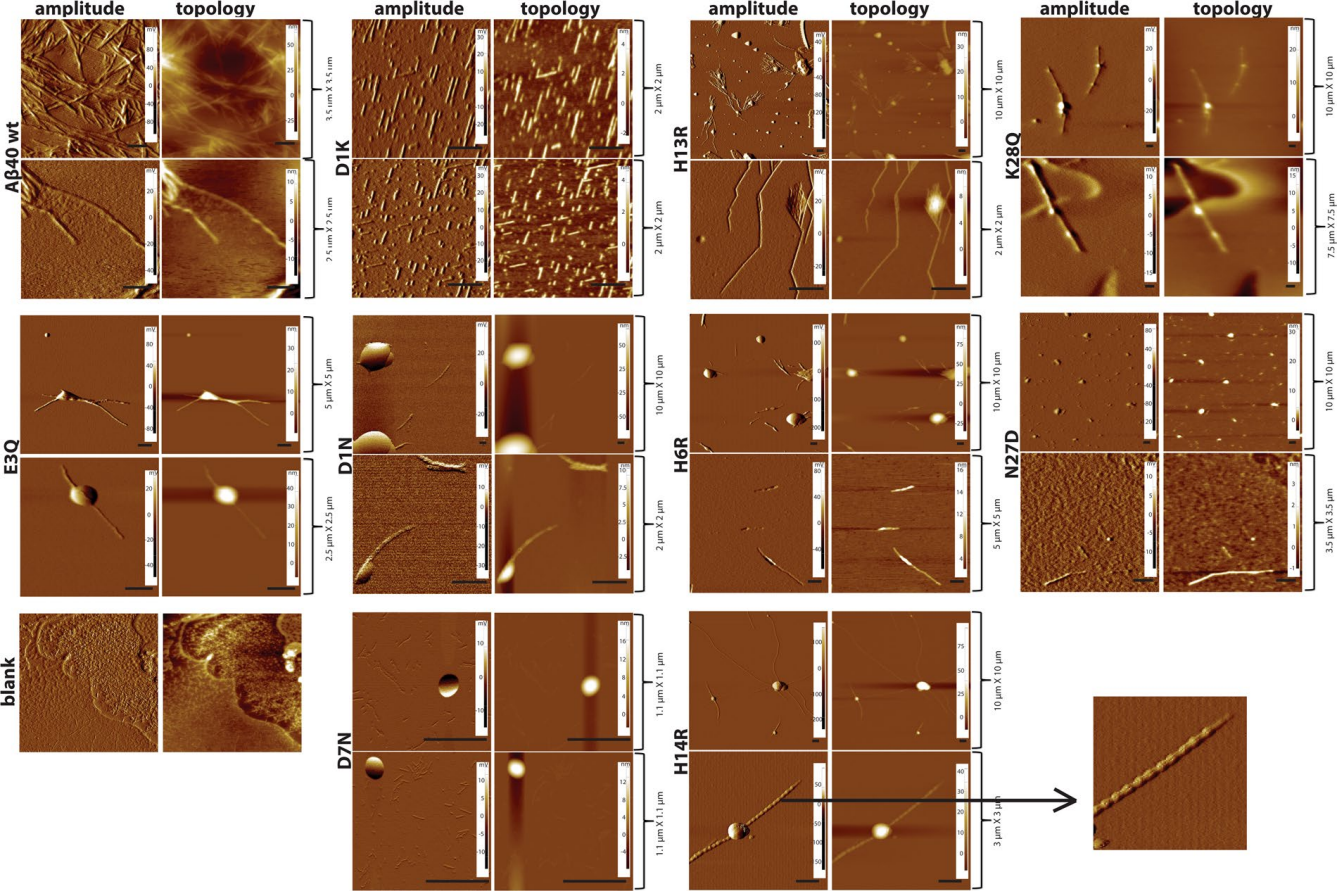
AFM images of fibrils formed by Aβ40 wild-type and mutants formed in 20 mM sodium phosphate buffer with 0.2 mM EDTA pH 7.4. Images of 256 × 256 pixels are shown and the scale bar corresponds to 0.5 µm. Fibril images are taken at different sizes as the length of fibrils varied between mutants. Amplitude and topology frames of each image are included.

To verify fibril formation, samples from the plateau phase of a reaction starting from 10 µM monomer were monitored using AFM (Fig. 7). Fibrils were observed for wild-type and all nine mutants examined. The fibrils of wild-type and eight of the mutants are between 0.2 and 1.7 µM (Table 2). Distinct periodic twists were observed for fibrils of two mutants (D1N: 0.05 µm and H14R: 0.25 µm) and the wild-type (0.1 µm). The N27D peptide barely showed any fibrils and contained spherical objects that appeared to be of two different sizes. The larger spherical particles resemble those seen in samples containing buffer and ThT only, suggesting that the smaller particles contain the peptide. When the N27D concentration was increased to 20 µM, both single fibrils and fibril clusters were detected.

**Table 2:** Length of the amyloid fibrils formed by Aβ40 and selected mutants.

Mutant	Length (µm)	Width (nm)
Wild-type	1.1 ± 0.4	55 ± 10
D1N	0.95 ± 0.3	70 ± 12.5
D1K	0.35 ± 0.1	70 ± 12.5
E3Q	3.3 ± 0.5	100 ± 30
D7N	0.3 ± 0.2	30 ± 10
H6R	1.3 ± 0.3	80 ± 20
H13R	1.3 ± 0.5	60 ± 25
H14R	3.3 ± 0.7	80 ± 20
K28Q	3.3 ± 1.05	200 ± 50
N27D*	0.75 ± 0.3	65 ± 10

To quantify the aggregation propensity over a broad concentration range, the aggregation half-time (t_1/2_) was extracted from the ThT data and plotted *versus* monomer concentration for each mutant (Fig. 6). The half-time will be shorter for the more aggregation prone variants. The trend of each of the mutant is compared to the wild-type on double logarithmic plots, log (t_½_) *vs* log (monomer concentration) (Fig. 6). The half-time plot of wild-type and all mutants except K28Q at higher concentration showed a change in scaling exponent indicating the saturation of aggregation kinetics within the range studied. D1K, D1N and D7N showed half-time curves below the wild-type at all concentrations, while for H13R it crossed the wild-type curve at higher concentrations indicating an increased level of saturation at higher concentrations. H6R, H14R and E3Q curves overlap with wild-type although E3Q flattens above the wild-type curve at higher concentrations. For K28Q and N27D, the half time is above that of the wild-type at all peptide concentrations. Clearly, mutants D1K, D1N, D7N and H13R tend to aggregate faster than wild-type over the whole or most of the concentration range. N27D, K28Q aggregate more slowly while H6R, H14R, E3Q show similar aggregation kinetics as the Aβ40 wild-type peptide. In Supplementary Fig. S1, the aggregation half-times of all mutants and wild-type are plotted in a single frame.

### Correlation between expression level and measure of aggregation rate

**Figure 8:**
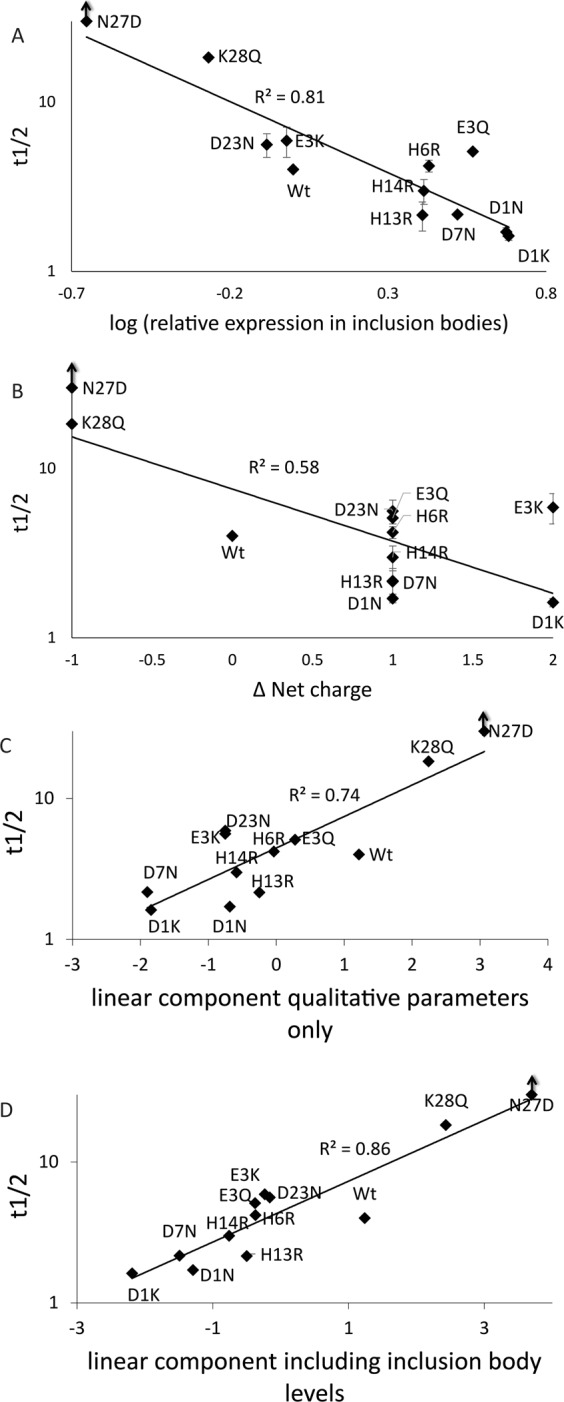
Plots showing the trends of: (**A**) half time vs relative expression of various Aβ mutant peptides in inclusion bodies (**B**) half time vs net charge of various Aβ mutant peptides, (**C**) half time vs linear principal component score based on all quantitative parameters, except inclusion body levels (**D**) half time vs linear principal component score based on all quantitative parameters including inclusion body levels. Note that logarithm y-axis is used and for x-axis log values of relative expression in inclusion bodies are used.

The strong correlation (R^2^ = 0.81) between the aggregation propensities (as inversely reported on by the half-time) of selected mutants and their expression in inclusion bodies is demonstrated in Fig. 8A where the half time of aggregation is plotted against the inclusion body formation keeping in mind that the reduced half-time reports on increased aggregation propensity. As a comparison the correlation with charge change is plotted in Fig. 8B (R^2^ = 0.58). To see if a stronger correlation could be obtained by including more parameters, the linear principal component calculated from all parameters (as described above for expression) without and with expression included (Fig. 8C,D). Notably the inclusion body expression together with other parameters increases the correlation further (R^2^ = 0.86) (Fig. 8C) illustrating that expression efficiency is the most important factor for predicting aggregation propensity (Fig. 8). Parameter loadings for 8 C and 8D are shown in Supplementary Fig. S2. Other than expression and charge, β-sheet propensity shows the largest loading.

## Discussion

Several studies have shown that mutations in Aβ may affect its rate of aggregation^8,17,22,38–46^. The aim of the present study was to examine whether the aggregation propensity of Aβ40 mutants correlates with the formation of inclusion bodies in *E*. *coli*. This hypothesis was based on the notion that inclusion-body formation may be considered to be an aggregation process.

Soluble unstructured peptides and small proteins may suffer from fast degradation in *E*. *coli*, preventing efficient recombinant production unless a protective tag is added. The high aggregation propensity of amyloid peptides rescues them from proteolysis and their formation of inclusion bodies in *E*. *coli* can be exploited as an advantage to enable tag-free expression of Aβ peptides^29^. Moreover, the low solubility of inclusion bodies allows for repeated sonication and removal of the majority of soluble *E*. *coli* proteins to obtain a suitable starting material for ion-exchange and size-exclusion isolation of Aβ with wild-type as well as mutant sequences^29^. Expression of amyloid proteins in *E*. *coli* is therefore fast, robust and inexpensive.

Although several factors influence the quality of expression, wild-type Aβ40 as well as most of the mutants were expressed at reproducible levels at the conditions used in this study. The amount of inclusion bodies did vary in a reproducible manner between different mutants as classified by SDS-PAGE analysis (Fig. 3). The amount of inclusion bodies is likely a reflection of the aggregation propensity relative to the degradation rate of soluble species rather than the expression level. Increased inclusion body formation is found for a set of mutants with a net charge that is less negative than for wild-type: D1K, D1N, E3Q, H6R, D7N, D7K, H13R, H14R and Q15K. Following this trend, we observe reduced inclusion body formation for all mutants with a net charge that is more negative: R5Q, Q15E, K16E, K16Q, N27D, and K28Q, some of which did not show any detectable peptide in inclusion bodies after overnight expression. Exceptions from the above patterns are the E3K and D23N mutants that show a reduced amount of inclusion bodies (which are less negatively charged than the wild-type) but also show slower aggregation than wild-type, suggesting that other factors than charge attenuate their inclusion body formation. In contrast to Aβ42, for which several labs have converged on the same structure^47–49^, a wide variety of Aβ40 fibril structures have been observed. However, in all Aβ40 fibril structures, both the E3 and D23 side chains are exposed to solvent, and in most cases D23 side chain is found on the surface of the ordered part of the fibril and E3 is in a more disordered region^50–55^.

The localization by CLSM of ThS-stained inclusion bodies in single bacterial cells showed a variation in the fluorescence intensities over the expressed mutants, which correlates with the amount of inclusion bodies as derived from the SDS-PAGE analysis. These results suggest the possibility to expand this system into a high-throughput technique for screening of a random library of Aβ peptide variants using techniques like fluorescent activated cell sorting (FACS). Earlier studies showed applications of FACS to screen for hydrolytic enzymes producing hydrogel-forming peptides in *E*. *coli* cells^56^. In the past, FACS has also been used in purification of amyloid proteins from brain tissues^57^. A low-throughput but detailed flow cytometry analysis has been reported for amyloid proteins expression in yeast cells and wild-type amyloid-β peptides in bacterial cells^58,59^. However, to our knowledge, screening the level of inclusion bodies of amyloid mutant libraries in bacterial cells, as an approach to find variants with altered aggregation propensity, has not been presented.

To test if the similar characteristics of proteins in inclusion bodies and in amyloid fibrils can be used to predict the rate at which proteins form amyloid fibrils we looked at the correlation between the rate at which proteins form amyloid fibrils and the amounts of inclusion bodies formed as characterized by SDS-PAGE and CLSM (Fig. 8). Our results show a correlation between the level of inclusion body formation and aggregation propensity, R^2^ of =0.81, is higher than obtained with any other parameters and further increased by inclusion of other parameters, suggesting the possibility of an approach to use inclusion body formation as proxy for aggregation propensity and another probe to further the understanding of factors modulating aggregation kinetics.

## Conclusions

In conclusion, we discovered a correlation between the charge of the Aβ40 mutants and the amount of inclusion bodies formed. The direction of the charge change relative to wild-type is more important than the amplitude of the change, i.e. less negatively charged mutants form higher levels of inclusion bodies, and more negatively charged mutants form lower amounts of inclusion bodies Furthermore, we see a strong correlation between inclusion bodies in *E*. *coli* and aggregation propensity in an *in vitro* aggregation kinetics assay for Aβ40 mutants. Thus mutants that form more inclusion bodies in *E*. *coli* also tend to aggregate faster and Aβ40 mutants that form less inclusion bodies tend to aggregate more slowly. Therefore, we propose that the method described in this study can be used as a preliminary screening technique to identify potentially more or less aggregation prone variants from screening of directed or random libraries for more in-depth kinetic analysis.

## Materials and Methods

### Expression of Aβ(M1-40) charge mutants

To be able to analyze the expression profile of all the charge mutants under typical growth conditions, four replicates of 500 mL cultures for each mutant as well as the wild-type was set up for overnight expression in auto inducing medium^60^. The Aβ (M1-40) peptides carry an N-terminal methionine residue to provide a start codon and are elsewhere referred to as Aβ40. The mutant plasmids (Pet3a with ampicillin resistance) were designed to each have a codon change leading to a single mutation at a hydrophilic positions of the Aβ40 peptide, and together the library members cover all such positions (Table S1). Acidic residues were changed to basic ones and *vice versa*. All plasmids were produced by GenScript (Piscataway, New Jesey). Chemically competent *E*. *coli* Bl21* plysS cells were used for transformation of the mutant plasmids and plated on LB agar plates with 50 µg/mL ampicillin and 30 µg/mL chloramphenicol. Single colonies were picked and each released into 50 mL LB medium containing 50 µg/mL ampicillin and 30 µg/mL chloramphenicol and allowed to grow to an OD between 0.6–1.8 at 125 rpm orbital shaking. Several pre-cultures were set up for each mutant from separate colonies, and 500 µL of each pre-culture was added to 500 mL of pre-warmed auto-inducing medium with the same antibiotics, which was grown for 15 h at 37 °C in 2.5 L baffled flasks at 125 rpm orbital shaking. 1 mL of each overnight culture was harvested and used for analysis of the expression profile of mutants by SDS-PAGE. The rest of each overnight culture was centrifuged (6000 g for 10 minutes) and stored at −20 °C for Aβ40 peptide purification.

### Cell disruption

Harvested cells from 1 mL of culture were suspended in 100 µl distilled water with 0.1% benzonase and incubated for 10 min at 37 °C. Cells were frozen at −80 °C for half an hour and thawed at room temperature for another half an hour. Freezing and thawing of cells was repeated five times and the samples were then centrifuged for 2 min at 13000 rpm. Each time, the supernatant was collected and the pellet was re-suspended in 100 µl of distilled water with 0.1% benzonase. The re-suspended solution was frozen at −80 °C overnight, thawed for half an hour and centrifuged for 2 min at 13000 rpm. Pellet was re-suspended in 50 µl of 8 M urea, incubated at room temperature for 30 minutes and further analyzed by SDS-PAGE.

### SDS-PAGE analysis

To compare the expression profiles and inclusion body formation of various mutants, the supernatant and the re-suspended pellet samples obtained after cell disruption were loaded on to the SDS-PAGE gel (see Supplementary Fig. S3). Samples were mixed in 1:1 ratio with sample buffer (0.5 M Tris HCl pH 6.8, 10% glycerol, 5% DTT, 0.05% bromophenol blue), incubated for overnight at room temperature and loaded on to a 10–20% gradient tricine gel (Invitrogen). The gel was run for 3 h at 80 V in 100 mM Tris, 100 mM tricine, 0.1% SDS, pH 8.3, stained in staining solution (0.25% Coomassie brilliant blue, 40% ethanol, 10% acetic acid, 50% water) for at least 4 h, destained in destaining solution (30% ethanol, 7% acetic acid) and finally scanned using imagescanner III, GE healthcare. BenchMark™ Low-range prestained protein standard from Novagen was used.

### Purification of the Aβ40 mutants

**Figure 9:**
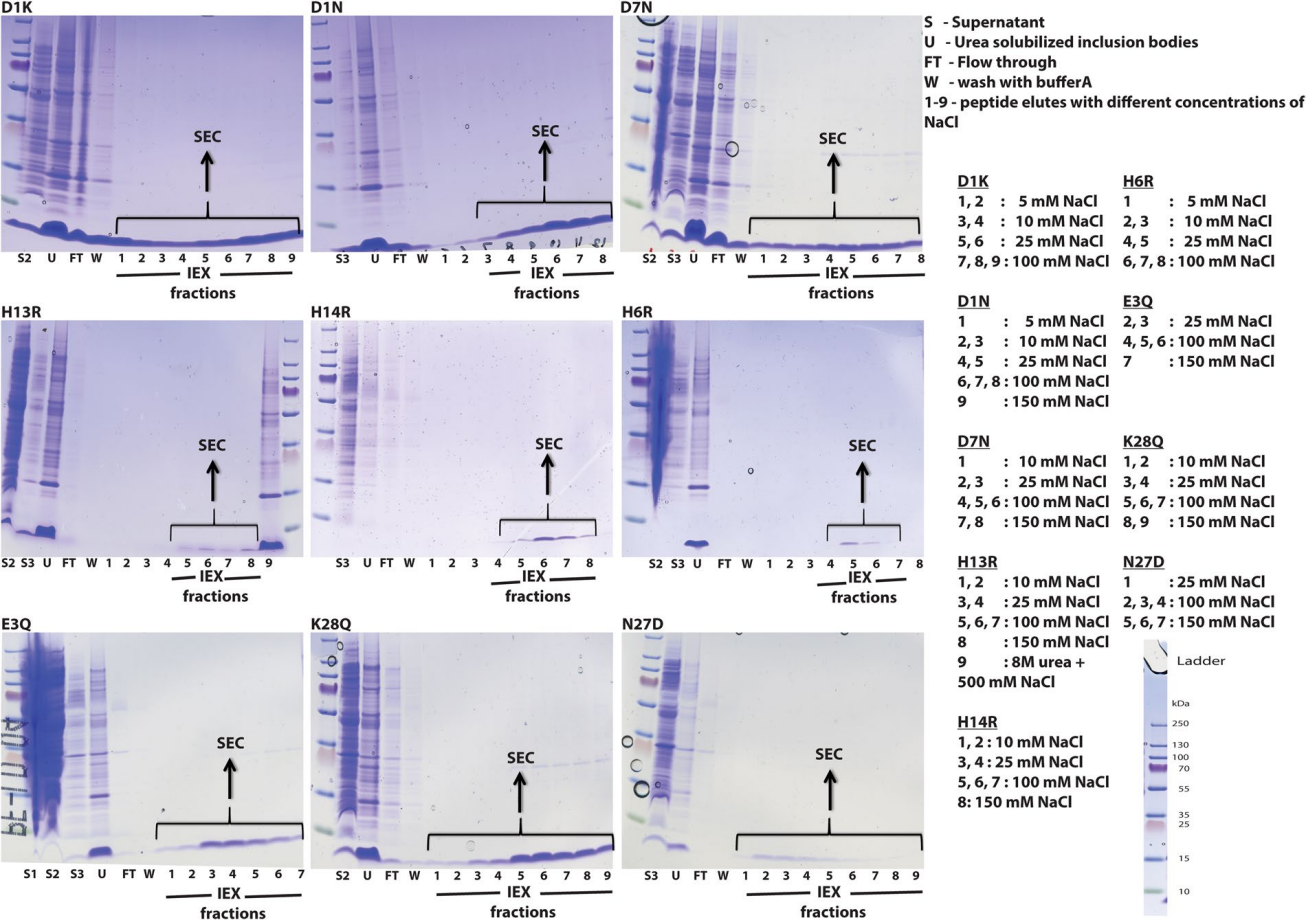
Ion exchange chromatography (IEX) of Aβ40 charge mutants. IEX fractions of each mutant are run on separate SDS-PAGE gel and labelled with respective mutation. The uncropped SDS-PAGE gels shown in Supplementary Fig. S4.

Frozen cell pellet from 2 L overnight culture was thawed on ice and re-suspended in 40 mL of 10 mM Tris/HCl pH 8.0, 1 mM EDTA (buffer A). The solution was sonicated twice for 1 min (50% duty cycle, strength 9.5, pulsed, ½ horn) on ice. Between sonications, the solution was cooled down on ice for 45 seconds. The solution was centrifuged for 10 min at 18000 × g, 4 °C in a JA 25.50 rotor, Avanti J-26 XP centrifuge. Supernatant was removed and pellet was re-suspended in 40 mL buffer A, sonicated two more times with centrifugation in between to remove supernatants. The final pellet was re-suspended in 20 mL ice cold 8 M urea, 10 mM Tris/HCl pH 8.0, 1 mM EDTA and sonicated to obtain a clear solution. The urea solubilized inclusion bodies were then 4-fold diluted with buffer A to obtain a final concentration of 2 M urea. Approximately 20 mL of DEAE-cellulose equilibrated in buffer A was added for purification via ion exchange chromatography. The slurry was carefully stirred with regular intervals for 20–30 minutes on ice. The slurry was poured in to a Büchner funnel with filter paper and placed on vacuum glass bottle to remove the filtrate. The resin was washed twice with 20 mL buffer A and followed by 20 mL portions of buffer A with 5 or 10 or 25 mM NaCl to remove weakly bound impurities. Low negative charge Aβ40 mutant peptides were eluted with buffer A with 100 mM NaCl and high negative charge Aβ40 mutants were eluted with buffer A with 150 mM NaCl. After eluting the protein, the resin was washed with 8 M urea 500 mM NaCl to elute all other proteins bound to the resin as a pre-cleaning before regeneration of the resin. Samples were collected at every filtration step and small aliquots were withdrawn for SDS-PAGE analysis (Fig. 9). Eluted Aβ40 peptides were filtered through a 30 kDa spin filter (Vivaspin 15 R, Saritorius Stedim Lab Ltd, UK) to remove proteases and other proteins that may digest or perturb the aggregation of amyloid beta peptide. An optional step of concentrating the peptide using 5 kDa spin filter was performed in some cases where the expression level of mutant peptide was low. Aliquot of 3–5 mL of peptide sample was lyophilized and stored at −20 °C until needed. A fresh gel filtration step was performed and center of monomer peak was collected and used immediately after collection to study aggregation kinetics of the peptide using ThT fluorescence as a reporter of fibril formation.

### *In vitro* aggregation kinetics of selected mutants

The freshly prepared monomer solution in 20 mM sodium phosphate buffer with 0.2 mM EDTA pH 7.4, as well as the same buffer without peptide, were supplemented with ThT at a final concentration of 20 µM and kept on ice. Dilution series of Aβ40 mutant peptides with concentrations ranging between 0.1–30, 0.1–50, 0.1–71 or 0.1–89 µM were prepared in low binding Eppendorf tubes (Genuine Axygen Quality, Microtubes, MCT-200-L-C). A 96-well PEG coated plate with a clear bottom (Corning 3881) was used to read the ThT fluorescence emitted from each mutant peptide sample. Each well was loaded with 100 μL of sample. Each mutant was studied in two different plates with triplicate samples for each concentration. (i.e. six replicates of each condition) The plate was sealed with a plastic film (Corning 3095). The plate was placed in a Polarstar Omega plate reader (BMG Labtech, Offenburg, Germany) and incubated at 37 °C without shaking. ThT fluorescence was measured every 2 s up to 120 h through the bottom of the plate, with the excitation and emission wavelengths at 440 and 480 nm, respectively. The half time (t_1/2_) was estimated by taking the values half-way in between start and end baseline.

### Analysis of the relationship between mutation-induced changes in physical properties and protein expression levels in inclusion bodies

Principal component analysis (PCA) was performed to assess the overall dependence of the amount of inclusion bodies, measured in SDS-PAGE bands using imageJ, or log (t_1/2_) for fibril formation on the mutation induced change in charge, helix propensity, β-sheet propensity, hydrophobicity and CamSol score using SIMCA15 (Umetrics, Malmö, Sweden). The resulting principal components were rotated to find the component that showed the strongest linear relationship with log inclusion body formation or with log (t_1/2_). In the latter case models were made both without and with log inclusion body formation included in the PCA.

### Mica surface preparation for AFM

AFM-grade mica in 11 mm × 11 mm pieces (agar scientific) and magnetized-stainless steel coin-like sample holders (12 mm diameter) were used. The mica pieces were glued on the top of the holder using double-sided sticky paper. Fibril samples were prepared from 10 µM or 20 µM monomer solutions. Identical protocol was followed for preparing Aβ40 mutant peptide samples as followed for sample preparation in kinetic analysis. Approximately 5 µL of sample was placed on each mica sheet and let to dry overnight by keeping it in a clean Petri dish.

### Atomic force microscopy

Images were obtained using an AFM instrument (Park systems, model 82) with the following settings: XY-scanner: 5 μm, 50 μm, or 100 μm; working distance of Z-scanner: 12 μm or 25 μm; optics of the AFM: direct on-axis vision of sample surface and cantilever; focus range: 20 mm; motorized magnification: 780x (optional 160x, 390x, or 1500x); field of view: 480 μm × 360 μm and optical resolution: 1 μm; AFM probes: non-contact mode, intermittent. Specifications of AFM probes: length- 125 µm, mean width- 30 µm, thickness- 4 µm, frequency- 330 kHz and force constant- 42 N/m.

### Confocal microscopy

Approximately 1 mL of bacterial cells expressing charge mutants were pelleted by centrifugation. Pellets were re-suspended and incubated for 1 h on ice in 0.3% Thioflavin S (ThS) solubilized in PBS and pelleted by centrifugation. Pellets were washed with PBS, centrifuged and re-suspended in PBS and placed on glass slide. Sample cells were mounted on an inverted CLSM (Leica TCS-SP5 tandem scanner), and imaged using a 100x oil immersion objective while in a thermostatic enclosure. Dye was excited with a 488 nm argon ion laser.

## Supplementary information

Supplementary file

## References

[1] Bucciantini M (2002). Inherent toxicity of aggregates implies a common mechanism for protein misfolding diseases. Nature.

[2] Ellis RJ, Pinheiro TJ (2002). Medicine: danger–misfolding proteins. Nature.

[3] Wisniewski T, Golabek AA, Kida E, Wisniewski KE, Frangione B (1995). Conformational mimicry in Alzheimer’s disease. Role of apolipoproteins in amyloidogenesis. Am J Pathol.

[4] Walsh DM (2002). Naturally secreted oligomers of amyloid beta protein potently inhibit hippocampal long-term potentiation *in vivo*. Nature.

[5] Ghiso J, Tagliavini F, Timmers WF, Frangione B (1989). Alzheimer’s disease amyloid precursor protein is present in senile plaques and cerebrospinal fluid: immunohistochemical and biochemical characterization. Biochem Biophys Res Commun.

[6] Anderson JP (1991). Exact cleavage site of Alzheimer amyloid precursor in neuronal PC-12 cells. Neurosci Lett.

[7] Mori H, Takio K, Ogawara M, Selkoe DJ (1992). Mass spectrometry of purified amyloid beta protein in Alzheimer’s disease. J Biol Chem.

[8] Storey E, Cappai R (1999). The amyloid precursor protein of Alzheimer’s disease and the Abeta peptide. Neuropathol Appl Neurobiol.

[9] Anderson JP, Chen Y, Kim KS, Robakis NK (1992). An alternative secretase cleavage produces soluble Alzheimer amyloid precursor protein containing a potentially amyloidogenic sequence. J Neurochem.

[10] Seubert P (1993). Secretion of beta-amyloid precursor protein cleaved at the amino terminus of the beta-amyloid peptide. Nature.

[11] Schonlein C, Probst A, Huber G (1993). Characterization of proteases with the specificity to cleave at the secretase-site of beta-APP. Neurosci Lett.

[12] Vassar R (2001). The beta-secretase, BACE: a prime drug target for Alzheimer’s disease. J Mol Neurosci.

[13] Wolfe MS (2001). Gamma-Secretase inhibitors as molecular probes of presenilin function. J Mol Neurosci.

[14] LaFerla FM, Green KN, Oddo S (2007). Intracellular amyloid-beta in Alzheimer’s disease. Nat Rev Neurosci.

[15] Hellstrand E, Boland B, Walsh DM, Linse S (2010). Amyloid beta-protein aggregation produces highly reproducible kinetic data and occurs by a two-phase process. ACS Chem Neurosci.

[16] Meisl G, Yang X, Dobson CM, Linse S, Knowles TPJ (2017). Modulation of electrostatic interactions to reveal a reaction network unifying the aggregation behaviour of the Abeta42 peptide and its variants. Chem Sci.

[17] Meisl G, Yang X, Frohm B, Knowles TP, Linse S (2016). Quantitative analysis of intrinsic and extrinsic factors in the aggregation mechanism of Alzheimer-associated Abeta-peptide. Sci Rep.

[18] Betts V (2008). Aggregation and catabolism of disease-associated intra-Abeta mutations: reduced proteolysis of AbetaA21G by neprilysin. Neurobiol Dis.

[19] Meisl G (2016). Molecular mechanisms of protein aggregation from global fitting of kinetic models. Nat Protoc.

[20] Abelein A, Jarvet J, Barth A, Graslund A, Danielsson J (2016). Ionic Strength Modulation of the Free Energy Landscape of Abeta40 Peptide Fibril Formation. J Am Chem Soc.

[21] Cohen, S. I. A. *et al*. *Nature Chemistry* (2018).

[22] Yang, X. M. G. On the role of sidechain size and charge in the aggregation of Aβ42 with familial mutations. *Proc Natl Acad Sci USA*, 10.1073/pnas.1803539115 (2018). 10.1073/pnas.1803539115 PMC6042101 29895690

[23] Przybycien TM, Dunn JP, Valax P, Georgiou G (1994). Secondary structure characterization of beta-lactamase inclusion bodies. Protein Eng.

[24] Georgiou G, Valax P, Ostermeier M, Horowitz PM (1994). Folding and aggregation of TEM beta-lactamase: analogies with the formation of inclusion bodies in Escherichia coli. Protein Sci.

[25] Carrio M, Gonzalez-Montalban N, Vera A, Villaverde A, Ventura S (2005). Amyloid-like properties of bacterial inclusion bodies. J Mol Biol.

[26] Garcia-Fruitos E (2005). Aggregation as bacterial inclusion bodies does not imply inactivation of enzymes and fluorescent proteins. Microb Cell Fact.

[27] Morell M (2008). Inclusion bodies: specificity in their aggregation process and amyloid-like structure. Biochim Biophys Acta.

[28] Achmuller C (2007). N(pro) fusion technology to produce proteins with authentic N termini in *E*. *coli*. Nat Methods.

[29] Walsh DM (2009). A facile method for expression and purification of the Alzheimer’s disease-associated amyloid beta-peptide. FEBS J.

[30] Ami D, Natalello A, Taylor G, Tonon G, Maria Doglia S (2006). Structural analysis of protein inclusion bodies by Fourier transform infrared microspectroscopy. Biochim Biophys Acta.

[31] Wang L, Maji SK, Sawaya MR, Eisenberg D, Riek R (2008). Bacterial inclusion bodies contain amyloid-like structure. PLoS Biol.

[32] Wang L (2009). Towards revealing the structure of bacterial inclusion bodies. Prion.

[33] Wurth C, Guimard NK, Hecht MH (2002). Mutations that reduce aggregation of the Alzheimer’s Abeta42 peptide: an unbiased search for the sequence determinants of Abeta amyloidogenesis. J Mol Biol.

[34] Bolognesi B (2014). Single point mutations induce a switch in the molecular mechanism of the aggregation of the Alzheimer’s disease associated Abeta42 peptide. ACS Chem Biol.

[35] Sormanni P, Aprile FA, Vendruscolo M (2015). The CamSol method of rational design of protein mutants with enhanced solubility. J Mol Biol.

[36] Sormanni P, Amery L, Ekizoglou S, Vendruscolo M, Popovic B (2017). Rapid and accurate in silico solubility screening of a monoclonal antibody library. Sci Rep.

[37] LeVine H (1993). Thioflavine T interaction with synthetic Alzheimer’s disease beta-amyloid peptides: detection of amyloid aggregation in solution. Protein Sci.

[38] Davis J, Van Nostrand WE (1996). Enhanced pathologic properties of Dutch-type mutant amyloid beta-protein. Proc Natl Acad Sci USA.

[39] Reaume AG (1996). Enhanced amyloidogenic processing of the beta-amyloid precursor protein in gene-targeted mice bearing the Swedish familial Alzheimer’s disease mutations and a “humanized” Abeta sequence. J Biol Chem.

[40] Robakis, N. K. & Efthimiopoulos, S. Familial Alzheimer disease: changes in Abeta production may indicate a disturbance in protein transport or function caused by pleiotropic effects of FAD mutations. *Neurobiol Aging* **20**, 81–83, discussion 87 (1999). 10.1016/s0197-4580(99)00011-1 10466898

[41] Siman R (2000). Presenilin-1 P264L knock-in mutation: differential effects on abeta production, amyloid deposition, and neuronal vulnerability. J Neurosci.

[42] Van Nostrand WE, Melchor JP, Cho HS, Greenberg SM, Rebeck GW (2001). Pathogenic effects of D23N Iowa mutant amyloid beta -protein. J Biol Chem.

[43] Nilsberth C (2001). The ‘Arctic’ APP mutation (E693G) causes Alzheimer’s disease by enhanced Abeta protofibril formation. Nat Neurosci.

[44] Grabowski TJ, Cho HS, Vonsattel JP, Rebeck GW, Greenberg SM (2001). Novel amyloid precursor protein mutation in an Iowa family with dementia and severe cerebral amyloid angiopathy. Ann Neurol.

[45] Yagi-Utsumi M, Dobson CM (2015). Conformational Effects of the A21G Flemish Mutation on the Aggregation of Amyloid beta Peptide. Biol Pharm Bull.

[46] Szczepankiewicz O (2015). N-Terminal Extensions Retard Abeta42 Fibril Formation but Allow Cross-Seeding and Coaggregation with Abeta42. J Am Chem Soc.

[47] Colvin MT (2016). Atomic Resolution Structure of Monomorphic Abeta42 Amyloid Fibrils. J Am Chem Soc.

[48] Walti MA (2016). Atomic-resolution structure of a disease-relevant Abeta(1–42) amyloid fibril. Proc Natl Acad Sci USA.

[49] Xiao Y (2015). Abeta(1–42) fibril structure illuminates self-recognition and replication of amyloid in Alzheimer’s disease. Nat Struct Mol Biol.

[50] Bertini I, Gonnelli L, Luchinat C, Mao J, Nesi A (2011). A New Structural Model of Aβ40 Fibrils. Journal of the American Chemical Society.

[51] Lu JX (2013). Molecular structure of beta-amyloid fibrils in Alzheimer’s disease brain tissue. Cell.

[52] Paravastu AK, Leapman RD, Yau WM, Tycko R (2008). Molecular structural basis for polymorphism in Alzheimer’s beta-amyloid fibrils. Proc Natl Acad Sci USA.

[53] Petkova AT (2005). Self-propagating, molecular-level polymorphism in Alzheimer’s beta-amyloid fibrils. Science.

[54] Petkova AT, Yau WM, Tycko R (2006). Experimental constraints on quaternary structure in Alzheimer’s beta-amyloid fibrils. Biochemistry.

[55] Olofsson A, Lindhagen-Persson M, Sauer-Eriksson AE, Ohman A (2007). Amide solvent protection analysis demonstrates that amyloid-beta(1–40) and amyloid-beta(1–42) form different fibrillar structures under identical conditions. Biochem J.

[56] Pitzler C (2014). A fluorescent hydrogel-based flow cytometry high-throughput screening platform for hydrolytic enzymes. Chem Biol.

[57] Palutke M, KuKuruga D, Wolfe D, Roher A (1987). Flow cytometric purification of Alzheimer’s disease amyloid plaque core protein using thioflavin T. Cytometry.

[58] Hidalgo IH (2016). Characterization of aggregate load and pattern in living yeast cells by flow cytometry. Biotechniques.

[59] Espargaro A, Villar-Pique A, Sabate R, Ventura S (2012). Yeast prions form infectious amyloid inclusion bodies in bacteria. Microb Cell Fact.

[60] Studier FW (2005). Protein production by auto-induction in high density shaking cultures. Protein Expr Purif.

