# Supplementary material for: A method of predicting the in vitro fibril formation propensity of Aβ40 mutants based on their inclusion body levels in E. coli
Source: Sci Rep. 2019 Mar 6;9:3680. doi: 10.1038/s41598-019-39216-z (PMC6403284; doi:10.1038/s41598-019-39216-z)
Supplement: Supplementary file 1 — Supplementary file [file 41598_2019_39216_MOESM1_ESM.pdf]

A method of predicting the *in vitro* fibril formation propensity of Aβ40 mutants based on their inclusion body levels in *E. coli*

Kalyani Sanagavarapu[1*], Elisabeth Nüske[2], Irem Nasir[3], Georg Meisl[4], Jasper N. Immink[5], Pietro Sormanni[4], Michele Vendruscolo[4], Tuomas P. J. Knowles[4,6], Anders Malmendal[1], Celia Cabaleiro-Lago[1,7], Sara Linse[1*]

1. *Lund University, Biochemistry and Structural Biology, Chemical Center, Lund, Sweden.*
2. *GEPADO-GmbH, Dresden, Germany.*
3. *Department of Integrative Structural and Computational Biology, The Scripps Research Institute, 10550 N. Torrey Pines Road, La Jolla, CA, 92037, USA.*
4. *University of Cambridge, Chemistry Department, Lensfield Road, Cambridge, UK.*
5. *Lund University, Physical Chemistry, Chemical Center, Lund, Sweden.*
6. *Cavendish Laboratory, Department of Physics, University of Cambridge, JJ Thomson Avenue, Cambridge UK.*
7. *Current address: Faculty of natural sciences, Kristianstad University, Sweden.*
8. ** Corresponding authors*

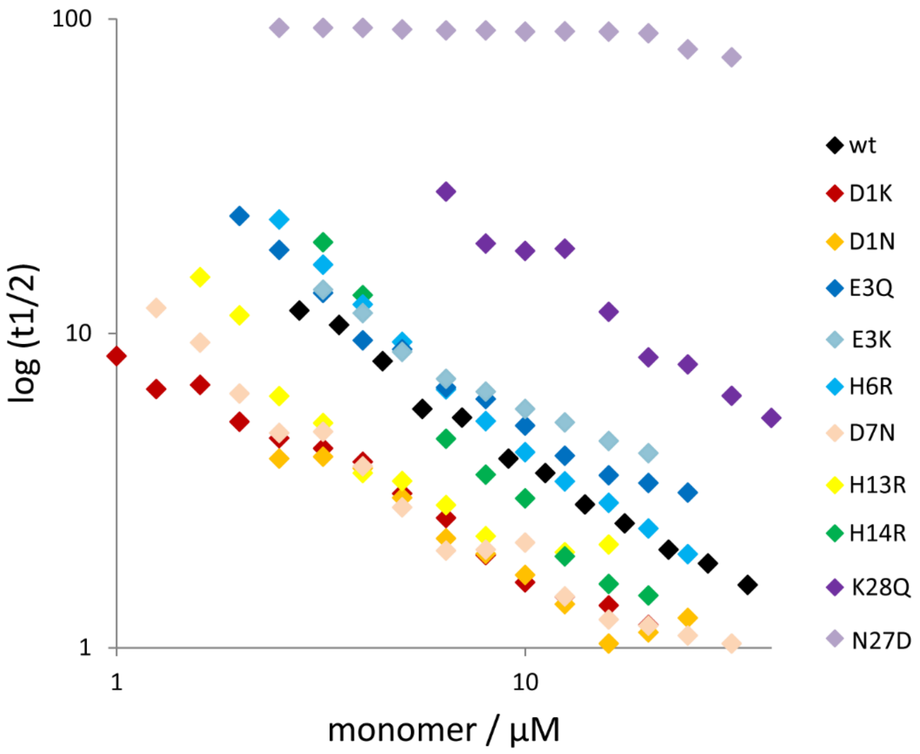

Supplementary Figure S1. Plot showing half-time of aggregation versus peptide concentration for Aβ40 wild-type (wt) and different charge variants (D1K, D1N, E3Q, E3K, H6R, D7N, H13R, H14R, K28Q and N27D).

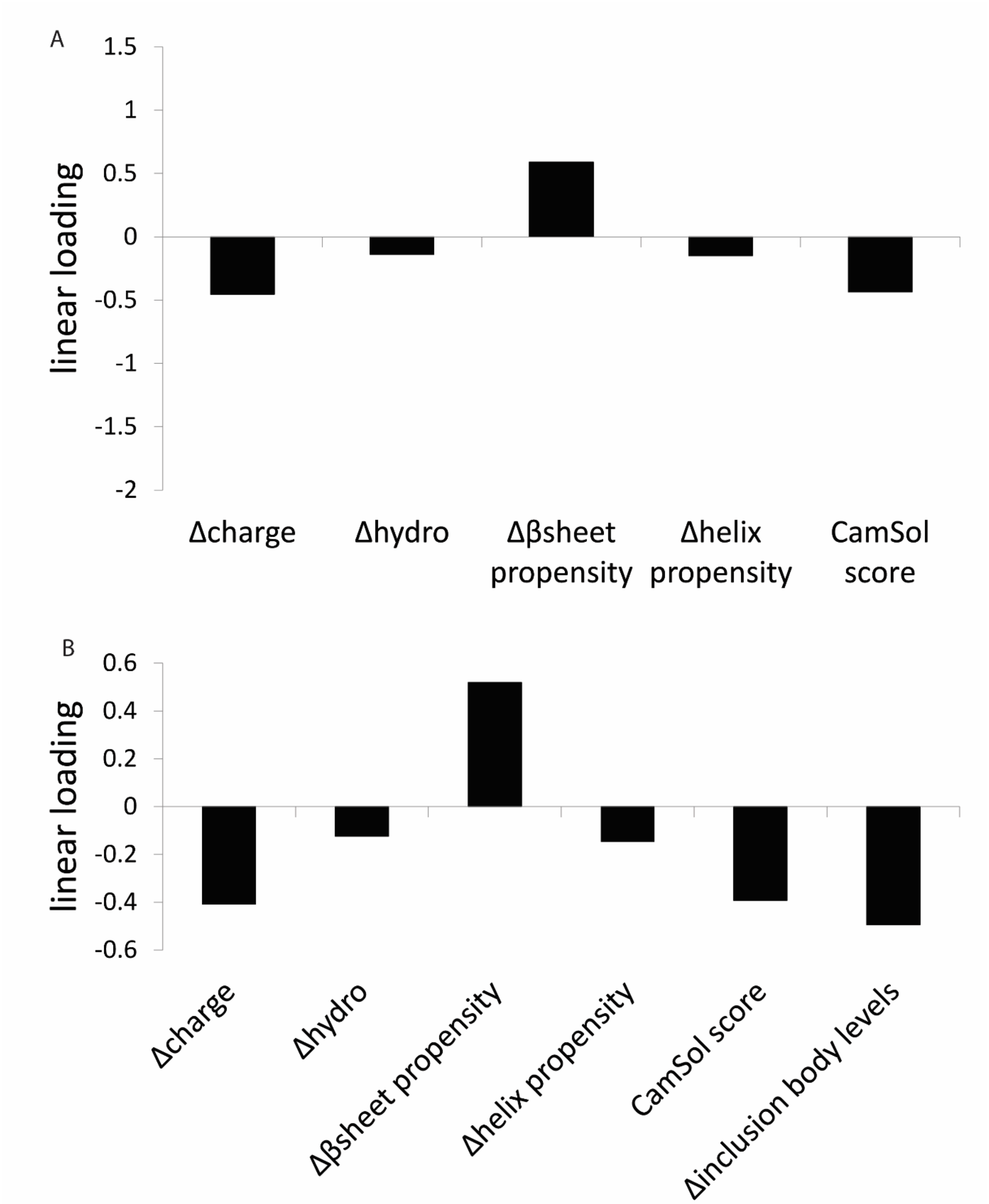

Supplementary Figure S2. Parameter loadings for linear components used in A. Figure 8C (without inclusion body levels) and B. 8D (with inclusion body levels).

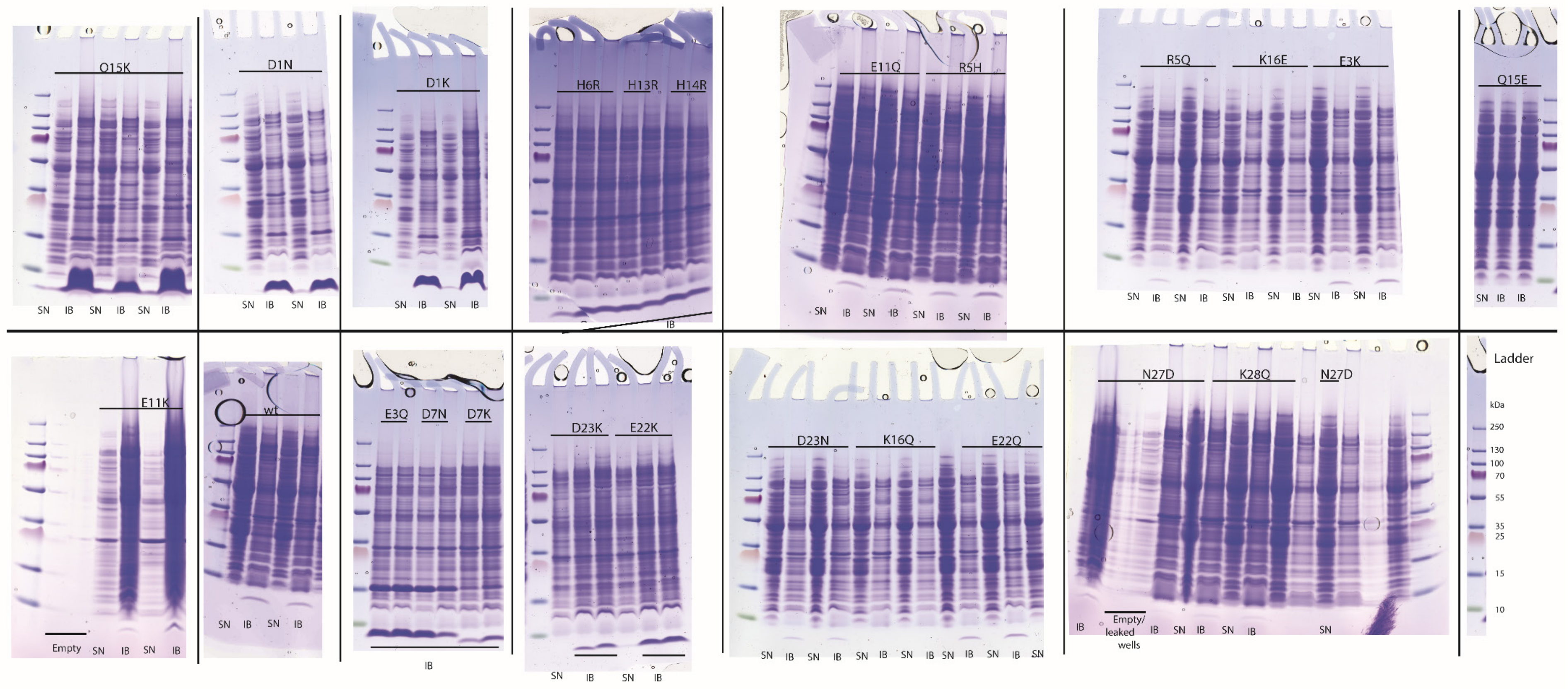

Supplementary Figure S3. SDS-PAGE analysis of inclusion body formation of Aβ40 charge mutants. Mutants were expressed on different days and were run on different SDS-PAGE gels. Duplicates of each sample were shown. Wells labelled with the mutation, lines indicate the wells included for each sample. Both supernatant (SN) and inclusion bodies (IB) were analyzed on SDS-PAGE gel. Gel bands shown in Figure 2 were cut from these gels.

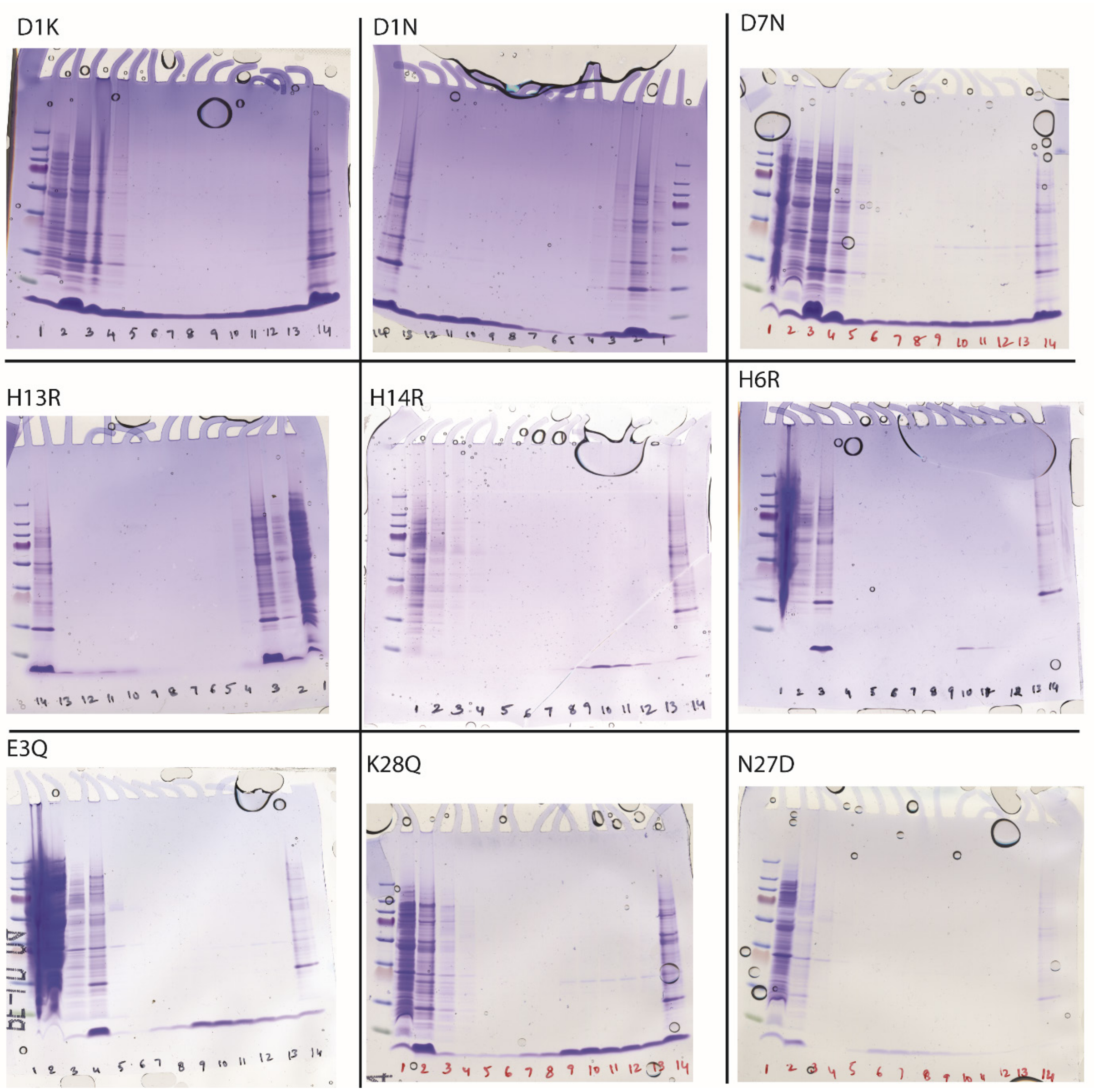

Supplementary Figure S4. Ion exchange chromatography of Aβ40 charge mutants. Uncropped SDS-PAGE gels for Figure 9. The details of samples in different wells is explained in Figure 9.

	Mutant	before	after	Δcharge	before	after	Δhydro	before	after	Δβsheet prop	CamSol score	pI
1	D1N	-3	-2	1	3.81	1.91	-1.9	0.72	0.40	-0.32	0.62	5.5
2	D1K	-3	-1	2	3.81	2.77	-1.04	0.72	0.34	-0.38	0.64	6
3	E3Q	-3	-2	1	2.91	1.30	-1.61	0.35	0.34	-0.01	0.57	5.48
4	E3K	-3	-1	2	2.91	2.77	-0.14	0.35	0.34	-0.01	0.62	5.99
5	R5H	-3	-4	-1	3.95	0.64	-3.31	0.35	0.37	0.02	0.59	5.03
6	R5Q	-3	-4	-1	3.95	1.30	-2.65	0.35	0.34	-0.01	0.60	4.74
7	H6R	-3	-2	1	0.64	3.95	3.31	0.37	0.35	-0.02	0.75	5.21
8	D7N	-3	-2	1	3.81	1.91	-1.9	0.72	0.40	-0.32	0.60	5.5
9	D7K	-3	-1	2	3.81	2.77	-1.04	0.72	0.34	-0.38	0.64	6
10	E11Q	-3	-2	1	2.91	1.30	-1.61	0.35	0.34	-0.01	0.60	5.48
11	E11K	-3	-2	2	2.91	1.30	-1.61	0.35	0.34	-0.01	0.66	5.99
12	H13R	-3	-2	1	0.64	3.95	3.31	0.37	0.35	-0.02	0.74	5.21
13	H14R	-3	-2	1	0.64	3.95	3.31	0.37	0.35	-0.02	0.79	5.21
14	Q15E	-3	-4	-1	1.30	2.91	1.61	0.34	0.35	0.01	0.82	4.83
15	Q15K	-3	-2	1	1.30	2.77	1.47	0.34	0.34	0	0.82	5.51
16	K16Q	-3	-4	-1	2.77	1.30	-1.47	0.34	0.34	0	0.46	4.74
17	K16E	-3	-5	-2	2.77	2.91	0.14	0.34	0.35	0.01	0.75	4.55
18	E22Q	-3	-2	1	2.91	1.30	-1.61	0.35	0.34	-0.01	0.43	5.48
19	E22K	-3	-1	2	2.91	2.77	-0.14	0.35	0.34	-0.01	0.60	5.99

20	D23K	-3	-1	2	3.81	2.77	-1.04	0.72	0.34	-0.38	0.61	6
21	D23N	-3	-2	1	3.81	1.91	-1.9	0.72	0.40	-0.32	0.53	5.5
22	N27D	-3	-4	-1	1.91	3.81	1.9	0.40	0.72	0.32	0.70	4.78
23	K28Q	-3	-4	-1	2.77	1.30	-1.47	0.34	0.34	0	0.59	4.74
24	K28E	-3	-5	-2	2.77	2.91	0.14	0.34	0.35	0.01	0.68	4.55

Supplementary Table S1. Aβ40 charge mutant peptides. Details of variation in charge, hydrophobicity and β sheet propensity before and after mutation while CamSol score and isoelectric point (pI) of each mutant calculated based on the sequence using protein pI calculator by Kozlowski LP, 2016 are included.
